# Dr. Milligan

**Published:** 1834-01-01

**Authors:** 


### OBITUARY.

Died, lately, at Edinburgh, in the fifty-third year of his age, Edward Milligan, m.d. He was born in a small village in Dumfries-shire, of poor but respectable parents, and received such an education as their humble circumstances would allow. At an early age he was apprenticed to a shoemaker, and continued to follow his trade until he had attained the age of manhood. He afterwards removed to Edinburgh, and worked at his trade; but, after a short time, studied Latin, and became a very good classic. Having made considerable progress in medical knowledge, he began teaching Latin to young men preparing for graduation, and by this means was enabled himself to graduate. It has been erroneously stated that he graduated only thirteen years ago; but, to the certain knowledge of the writer of this imperfect sketch, Dr. Milligan was already an m.d. in 1817. As soon as he had obtained the degree of m.d. he became a regular private tutor, and in this capacity was highly esteemed, not only by the young men under his care, but he enjoyed the respect and friendship of those who had formerly profited by his instruction. He was indefatigable in his endeavors to convey information to those who were willing to receive it.

During the winter session of the Edinburgh classes, he constantly rose at six o'clock in the morning to receive his pupils, and was in this way fully occupied until midnight, allowing himself but very short intermissions for his meals. He was thus enabled to amass some property. About five or six years since he was deprived of his sight by amaurosis, after which he declined receiving any more pupils.

He was the translator of Magendie's Physiology, and the editor of an extremely accurate and excellent edition of Celsus. In 1819 and 1820, he lectured in Edinburgh on Physiology, and had a tolerably good class. In 1820 he became joint editor of the Edinburgh Journal of Medical Science, which was most excellent both in its arrangement and contents, and promised fair to have had an extensive circulation; but some disagreement occurred amongst the editors, and, after living a year and a half, the publication came to a premature end.

Such was Dr. Milligan; a striking example of the valuable truth, that unremitting industry, even when unaccompanied by brilliant talents, is certain to lead to competence and content.

---

